# Inclusion of bioclimatic variables in genetic evaluations of dairy cattle

**DOI:** 10.5713/ajas.19.0960

**Published:** 2020-05-12

**Authors:** Renata Negri, Ignacio Aguilar, Giovani Luis Feltes, Juliana Dementshuk Machado, José Braccini Neto, Fabiana Martins Costa-Maia, Jaime Araújo Cobuci

**Affiliations:** 1 Department of Animal Science, Federal University of Rio Grande of Sul, Porto Alegre, RS, 91540-000, Brazil; 2 Instituto Nacional de Investigación Agropecuaria (INIA), Rincón del Colorado, CA, 90200, Uruguay; 3 Department of Animal Science, Federal Technological University of Paraná, Dois Vizinhos, PR, 85660-000, Brazil

**Keywords:** Heat Stress, Random Regression, Temperature-humidity Index, Diurnal Temperature Variation

## Abstract

**Objective:**

Considering the importance of dairy farming and the negative effects of heat stress, more tolerant genotypes need to be identified. The objective of this study was to investigate the effect of heat stress via temperature-humidity index (THI) and diurnal temperature variation (DTV) in the genetic evaluations for daily milk yield of Holstein dairy cattle, using random regression models.

**Methods:**

The data comprised 94,549 test-day records of 11,294 first parity Holstein cows from Brazil, collected from 1997 to 2013, and bioclimatic data (THI and DTV) from 18 weather stations. Least square linear regression models were used to determine the THI and DTV thresholds for milk yield losses caused by heat stress. In addition to the standard model (SM, without bioclimatic variables), THI and DTV were combined in various ways and tested for different days, totaling 41 models.

**Results:**

The THI and DTV thresholds for milk yield losses was THI = 74 (−0.106 kg/d/THI) and DTV = 13 (−0.045 kg/d/DTV). The model that included THI and DTV as fixed effects, considering the two-day average, presented better fit (−2logL, Akaike information criterion, and Bayesian information criterion). The estimated breeding values (EBVs) and the reliabilities of the EBVs improved when using this model.

**Conclusion:**

Sires are re-ranking when heat stress indicators are included in the model. Genetic evaluation using the mean of two days of THI and DTV as fixed effect, improved EBVs and EBVs reliability.

## INTRODUCTION

Brazil is the fourth largest milk producer in the world, with an annual milk production of 33.5 billion liters [1]. Minas Gerais is historically the largest dairy region in the country, accounting for 27% of the national production. However, the production potential may be threatened due to heat stress that negatively impacts livestock production, especially in tropical regions [2,3].

Traditionally, dairy cattle breeding programs have focused on intense selection to increase milk yield. However, it has already been proven that milk yield and heat tolerance are antagonistically correlated [4–7], and intense selection for milk production increases sensitivity to heat stress [7]. To minimize this effect, it is necessary to quantify heat stress, correct the data for the effect and, than carry out the genetic evaluation. Failure to include heat stress indicators can affect the estimation of breeding values (EBVs), compromising selection.

The methodology for including bioclimatic variables to quantify the level of heat stress has shown positive results in animal selection in subtropical regions [2,3,5,7]. Heat stress is diagnosed by decreasing the daily milk production after a specific limit of the bioclimatic indicator. The most used indicators to determine the degree of heat stress in dairy cattle are temperature-humidity index (THI) and diurnal temperature variation (DTV). THI is a unitary bioclimatic index that represents a combination of temperature and air humidity, while DTV is obtained by the difference between maximum and minimum daily temperatures.

Despite many researches on dairy cattle, little is known about the implication of data correcting considering bioclimatic indicators of heat stress and what is the best way to correct the data for this effect and its implications for the re-ranking of animals. Considering the importance of dairy farming in Brazil and the negative effects of heat stress on almost all livestock activities, investigating factors that affect yields is necessary to producers to improve their herd quality and become competitive in the market.

In this context, the objective of this study was to investigate the effect of heat stress via THI and DTV in the genetic evaluations for daily milk yield of Holstein dairy cattle, using random regression models.

## MATERIALS AND METHODS

### Data

Data consisted of test-day (TD) milk yield collected by the Service of the Minas Gerais Association of Holstein Breeders (ACGHMG), accredited by the Ministry of Agriculture, Livestock and Supply (MAPA). Data of Holstein cows of the state of Minas Gerais – Brazil (19°55′ S – 43°57′ W), from 1996 to 2015 were used. For this study, only information from the first lactation was considered. We excluded records with extreme age at calving (<18 or >48 months), days in milk (DIM, <5 or >305 days) and milk yield (<4 or >44.8 kg) from the data set. Only healthy animals with at least four individual TD records during lactation were retained for analysis. The minimum size of each contemporary group (described in each model) was three animals. Records of daughters of sires with at least one daughter in at least three herds were accepted to the evaluation.

Following these criteria, a total of 94,549 TD records from 11,294 first lactations of Holstein cows (average calving age: 29 months) from 129 farms, collected from 1997 to 2013, were analyzed. The same database was used for all models evaluated. The pedigree file included 32,409 animals. The edited data are described in Table 1.

Minas Gerais is characterized by three predominant climate types (according to the Köppen-Geiger climate classification): subtropical of altitude; subtropical with dry winter; and tropical with dry winter. Seasonal factors influence the herd management, in warmer conditions the animals tend to be kept in pastures, while in colder periods animals are semi-confined and supplemented with silage [8].

**Figure 1:**
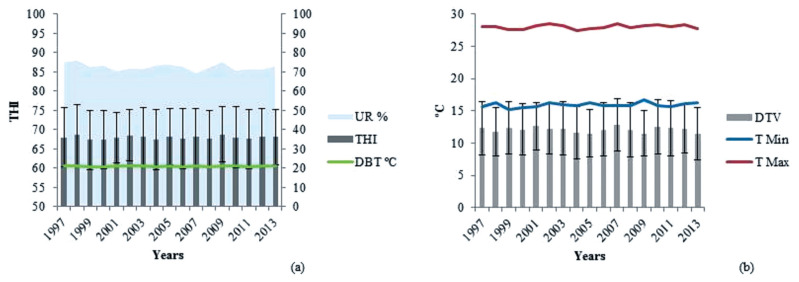
Annual average bioclimatic information: Dry bulb temperature (DBT, °C), relative humidity (UR, %) and temperature-humidity index (THI) (a); diurnal temperature variation (DTV, °C), maximum temperature (T Max, °C) and minimum temperature (T Min, °C) (b).

The climate variables used were average daily dry bulb temperature (DBT; °C), maximum temperature (°C), minimum temperature (°C), and average daily relative humidity (RH; %) (Figure 1), as recorded by the National Institute of Meteorology through 18 weather stations (representing 86 municipalities) located less than 60 km away from the evaluated farms, using the nearest station information [9]. The THI was evaluated according to equation described by the National Research Council [10]:

THI=(1.8×DBT+32)-[(0.55-0.0055×RH)×(1.8×DBT-26)]

The DTV was calculated by the difference between daily maximum and minimum temperatures, in degrees celsius (°C).

### Models

**Figure 2:**
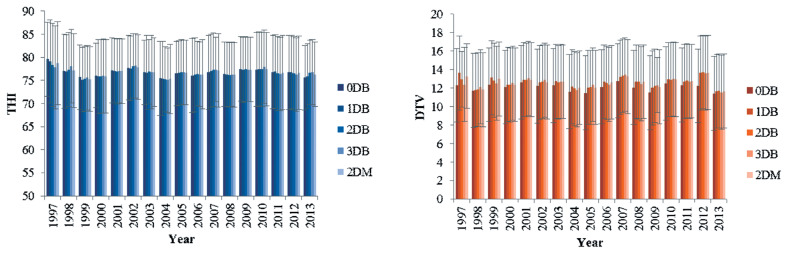
Average estimates of the temperature-humidity index (THI) and diurnal temperature variation (DTV) according to the evaluation day: THI and DTV on the test-day (TD) record (0DB); one day before (1DB); two days before (2DB); three days before (3DB), and mean between the two last days before each TD (2DM).

The daily averages of THI and DTV were tested up to three days before each TD record - THI and DTV on the TD record (0DB); one day before (1DB); two days before (2DB); three days before (3DB), and mean between the two last days before each TD (1DB and 2DB) (2DM) (Figure 2). It was not possible to test more days before the control because of the lack of THI and DTV for some herds, which would increase the elimination of data in approximately 40% due to the need to use the same data file for all models.

To delimit the heat comfort zone, the average loss of daily milk yield per THI and DTV unit was estimated by linear regression of milk yield on the THI and DTV values as deviation from the threshold limit. Fixed effects of the threshold model were considered: contemporary group, milking frequency, and DIM. The age of cows at calving was considered covariate (linear effect). The average daily loss of milk yield was estimated considering the mean THI and DTV values (only 2DM).

For genetic evaluation, a random regression model was used for the TD milk yield analysis using the Wilmink parametric function [11]. The additive genetic and permanent environmental covariance functions were estimated by the random regression model of the DIM for 9 models, which will be defined later (standard model [SM], M1, M2, M3, M4, M5, M6, M7, and M8).

The standard random regression model does not consider bioclimatic variables for correction of the data, as shown by the equation:

$$(\text{SM})\;y_{ijklm}={HYM}_{l}+\sum_{m=0}^{df}\varphi_{jkm}\beta_{m}+\sum_{m=0}^{dr}\varphi_{jkm}\mu_{jm}+\sum_{m=0}^{dr}\varphi_{jkm}pe_{jm}+e_{ijklm}$$

where *y_ijklm_* is the *i*th TD record of the *j*th cow on the *k*th DIM within the *l*th subclass herd-year-month of the test (HYM); *β_m_* is the *m*th fixed regression coefficient, defined as the age classes: 1 (18 to 25 months), 2 (26 to 27 months), 3 (28 to 29 months), and 4 (30 to 48 months), combined with the calving season subclasses: 1 (rainy: October to March) and 2 (dry: April to September), totaling eight fixed curves; *μ_jm_* is the *m*th random regression coefficient for the additive genetic effect of the *j*th cow; *pe_jm_* is the *m*th random regression coefficient for the permanent environmental effect the *j*th cow; *φ_jlm_* is the *m*th Wilmink function corresponding to the TD record of the *k*th DIM of the *j*th cow; *df* and *dr* are orders of fixed and random regression coefficients; and *e_ijklm_* is the random residual effect.

The M1 to M8 models were used to verify the best way to include bioclimatic data for the correction of the genetic evaluation model, according to the day included with THI and DTV data: 0DB, 1DB, 2DB, 3DB, and 2DM, totaling 40 models.

Model 1 (M1): Contemporary group and THI class (21 classes, every two units of THI was considered a class: THI 51 and 52 = class 1, THI 53 and 54 = class 2,…, THI 91 and 92 = class 21) (fixed effects).
Model 2 (M2): Contemporary group (fixed effect), and THI (linear covariate).
Model 3 (M3): Contemporary group (fixed effect), and THI (linear and quadratic covariates).
Model 4 (M4): Contemporary group (fixed effect), and DTV (linear covariate).
Model 5 (M5): Contemporary group is defined as herd-THI class (twenty-seven THI classes) (fixed effect). Only model with a contemporary group different from that defined in SM.
Model 6 (M6): Contemporary group (fixed effect), THI and DTV (linear covariates).
Model 7 (M7): Contemporary group, THI and DTV (fixed effects).
Model 8 (M8): Contemporary group (fixed effect).

The fixed curve is defined as age classes: 1 (18 to 25 months), 2 (26 to 27 months), 3 (28 to 29 months), and 4 (30 to 48 months), combined with the THI subclasses (seven classes, with five THI each class: THI 51 to 56 = class 1, THI 57 to 62 = class 2, …, THI 87 to 92 = class 7), totaling twenty-eight fixed curves. Only model with a fixed curve different from that defined in SM.

For all models, the fixed curve, additive genetics and permanent environmental covariance functions were estimated by random regression used Wilmink of DIM. The residual variance was considered homogeneous for all models.

### Genetic evaluation and statistical analysis

All genetic analyses were performed with an animal model, using the REMLF90 program [12]. The quality of the adjustment was carried out through comparison tests between non-nested models and penalties according to the number of parameters to be estimated. The following criteria were used: log-likelihood function (−2logL); Akaike’s information criterion (*AIC* = −*2logL*+*2p*, where *p* is the number of parameters in the model); Schwarz’s Bayesian information criterion (*BIC* = −*2logL*+*p*log(λ), where log (λ) is the natural logarithm of the sample size (or dimension of *y*) and p is the number of parameters in the model), BIC is more rigid than AIC. The model with the lowest value, for both criteria, is considered the best fit. To check the re-ranking of the estimated breeding values (EBVs) of the sires, Spearman rank correlation coefficient (*p*) for 1% and 10% of the upper sires EBVs was used. The reliabilities of the EBVs were calculated using the triangular matrices of prediction error (co)variances for random regression effects, from the inverse of the mixed model equations obtained in the BLUPF90 program [12].

## RESULTS AND DISCUSSION

### Milk yield

Climatic conditions were found to exert an influence on milk production in dairy farms in Minas Gerais State, Brazil. The bioclimatic variables used as indicators of heat stress play an important role in animal production, predicting the critical limit between comfort and stress. The identified thresholds provide the essential pre-requisites for identification of genetic components of heat stress and allow promote solutions and the development of improvement strategies.

THI is the most widely used environmental indicator of heat stress effects in literature. However, DTV also has the potential to correct data and minimize the stressor effect on genetic evaluations.

**Figure 3:**
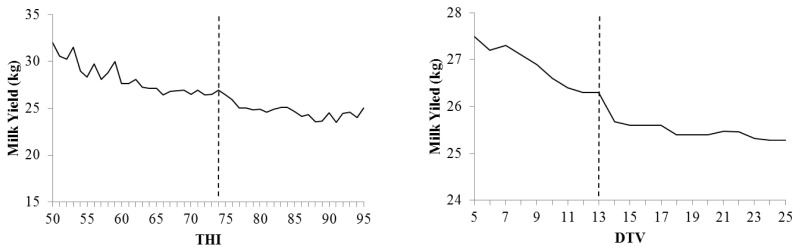
Heat stress threshold (dotted line) and average daily milk yield corrected according to temperature-humidity index (THI = 74) and diurnal temperature variation (DTV = 13).

The THI threshold for milk yield losses was THI = 74, considering the complete lactation (5 to 305 day) (Figure 3). The decreases in milk production were −0.106 kg/cow/d/THI unit above 74. The effects of THI stratified according to lactation phase (initial, DIM 5 to 60; intermediate, DIM 61 to 180; and final phase, DIM 181 to 305) showed a significant effect of THI on milk yield, with decreases of −0.092 kg/d/THI (DIM 5 to 60, p<0.0001), −0.108 kg/d/THI (DIM 61 to 180, p<0.0001), and −0.114 kg/d/THI (DIM 181 to 305, p<0.0001).

The DTV threshold was DTV = 13. The average annual temperature of the state of Minas Gerais is approximately 16.3°C, with average daytime temperature variation of 5.8°C to 7.6°C [9]. This thermal amplitude above 13°C causes decreases in milk yield of cows by up to −0.045 kg/DTV unit (p<0.001) increased above 13. The effects of DTV stratified according to the lactation phase were significant for milk yield, with decreases of −0.002 kg/d/DTV (DIM 5 to 60, p = 0.002), −0.056 kg/d/DTV (DIM 61 to 180, p = 0.004), and −0.005 kg/d/DTV (DIM 181 to 305, p = 0.003).

This study, most cows are daughters of sires that imported semen (especially from the United States, Canada, and the European Union). Approximately 90% of lactating cows were exposed to heat stress conditions in at least five TD records; and 69% of the TD records were in conditions of heat stress. This result is concerning due to potential of economic impacts. The loss of 1.28±0.31 kg of milk at the peak of lactation by heat stress reflects a loss of production of about 221±2.2 kg at the end of complete lactation; however, this result can reach 2,000 kg of loss. In addition, cows that calved in the summer (average THI = 82 and DTV = 14) produced an average of 6% less milk when compared to cows calved in the beginning of winter (average THI = 73 and DTV = 10).

The magnitude of production losses shows the importance of evaluating heat stress through bioclimatic variables, especially when considering the economic factor. The impact on reducing milk production is very expensive, and this scenario can be extrapolated to Brazil and world, because the climate of Minas Gerais represents a large part of the Brazilian climate and of several regions of the world.

Listed as one of the major concerns in dairy cattle, heat stress affects production potential almost worldwide and it is believed that the financial impact due to heat stress probably outweighs the impact due to mastitis and reproductive parameters. In addition, the combination of elevated temperature and humidity negatively affects quality milk, food intake [10], and reproductive potential [13]. When it comes to Holstein cattle, the values quoted in the literature are similar to the result obtained in these studies, in Missouri the estimated threshold was THI = 70 [14], in Georgia THI = 72 [15] and, in Arizona THI = 74 [5]. In Thailand, Sae-tiao et al [16] estimated decreases of −0.029 kg/cow/d due to DVT (°C). DTV also negatively affects milk production, but to a lesser extent, when compared to THI, however, the variable deserves attention in daily management to minimize the effects.

### Adjustment of models

**Figure 4:**
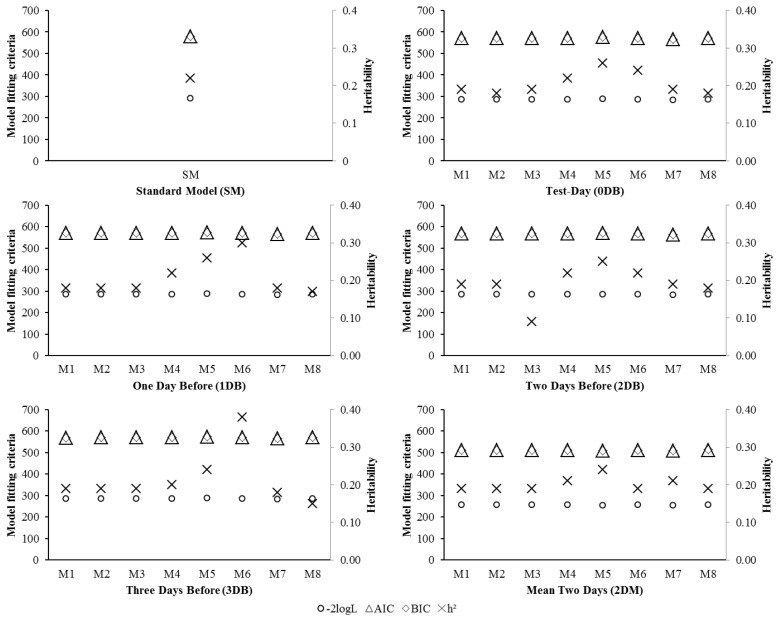
Estimates of models fitting criteria: log-likelihood function (−2logL), Akaike information criterion (AIC) and Schwarz Bayesian information criterion (BIC); and heritability estimates for 305 days of milk production (h^2^), for each model (M1 to M8) and day of inclusion of bioclimatic variables.

Most models that consider bioclimatological variables present better fit than the SM (Figure 4). M7 included THI and DTV as a fixed effect and presented the best overall fit for correcting milk yield data, regardless of the day included with THI and DTV data. The M7-2DM was the best adjustment model, denoting that the mean between 1DB and 2DB better explains the milk yield loss due to heat stress.

Biologically, the use of 2DM of stress-causing factors (THI and DTV) can better explain the animal performance due to the amount of circulating cortisol in the body. Result that agrees with that obtained by West et al [17], who stated that the use of weather information, as in the 2DM, better shows the effect of heat stress on milk yield. The response to heat stress is not immediate, but cumulative [13]. The plasma cortisol concentration in animals exposed to heat stress shows a peak in the first 12 hours after the onset of heat stress and tends to return to normal values within two days [18]. Thus, the environment is important for metabolism and affects the cardiovascular system and absorption of nutrients in the mammary gland, directly affecting milk production [19].

To check the impact of heat stress on the estimation of sires EBVs, in addition to the SM (routinely used), the best model for each day tested was chosen to verify the change in the sires’ rank: M7-0DB, M7-1DB, M7-2DB, M7-3DB, and M7-2DM.

### Estimated breeding values, ranking and reliability

**Figure 5:**
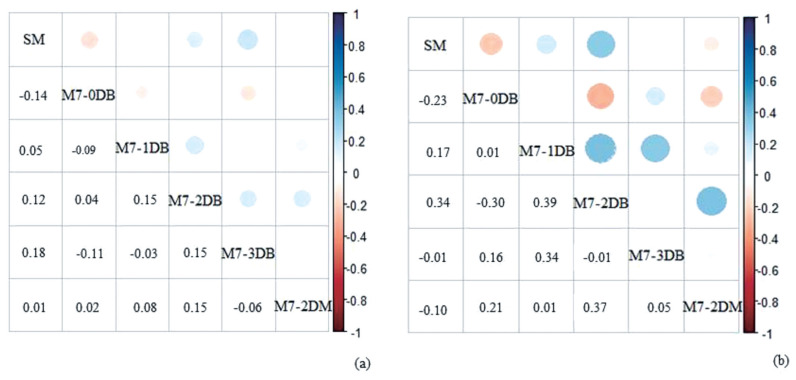
Spearman rank correlations coefficient for the top 10% (a) and top 1% (b) best sires (estimated breeding values) for milk yield, according to the standard model (SM). SM and best adjusted model (M7) for the days included with temperature-humidity index and diurnal temperature variation data—on test-day record day (M7-0DB); one day before (M7-1DB); two days before (M7-2DB); three days before (M7-3DB), and mean between the two last days before (M7-2DM). Positive correlations are displayed in blue and negative correlations in red color. Color intensity and the size of the circle are proportional to the correlation coefficients.

The magnitude of the estimated Spearman rank correlations coefficient, especially for the SM, confirmed the reranking of the sires when including the bioclimatic variables in the models (Figure 5). The EBVs of the animals changed when correcting the data for the stress indicators, so the selection process may be compromised and the observed genetic gains may not be equal to the expected genetic gains. Genetic superiority animals may be being eliminated from the mating selection due to the fact that their daughters are more penalized for disregarding heat stress in the evaluation.

This probably affects not only the producer, but the entire dairy industry. Research shows significant economic losses of around $900 million dollars per year [20]. St-Pierre et al [21] estimated an annual economic loss of $897 million to $1.5 billion dollars for the USA dairy industry due to the heat stress of dairy cattle.

In Brazil, the strategy of farmers in the search for productivity improvement is the use of imported semen. It is genetic material selected in other countries under different environmental conditions. However, the milk production of these animals in Brazil is not expected to correlate with those in the environments in which they were originally bred. In addition to milk yield, other traits and parameters of milk quality are also highly affected by bioclimatic features [22].

According to Zwald et al [23], the genotype-environment interaction can significantly affect productive and reproductive characteristics, causing re-rankings; and variables, such as temperature, can be used to group herds into similar production environments. Robertson [24] reported that the re-ranking can be aggravated by combining different characteristics into a selection index, since genetic correlations below 0.8 may result in re-ranking of the animals. The same was found in South Korean dairy cattle by Lee et al [25], where selection for high milk yield decreased thermotolerance and when the THI incorporated, the sires ranking was changed. The re-ranking and changes in the magnitude of differences of the genetic merit of animals can affect important productive and economic aspects.

The comparison of the reliability of the EBVs of the ten best sires and the top 1% for milk yield in the TD records in the SM with the reliability of the EBVs of these sires in the other models evaluated, showed no changes, although there was a re-ranking (Table 2). However, when extrapolating to the top 5%, top 10%, and to all sires evaluated, a significant difference (p<0.05) was found when considering the fixed effects in the M7-2DM model to estimate the reliability of the EBVs of the sires (Table 2). The mean reliability of the EBVs of the sires for the milk yield in TD record was 10% to 25% higher for the top 5%, 18% to 35% higher for the top 10%, and 75% to 100% for all sires evaluated, when using the M7-2DM model.

Sires with few daughters had lower reliability of the EBVs when using the SM (Table 3). When included the two day averages of THI and DTV as a fixed effect in the genetic evaluation model, it’s possible to observe an increase in reliability of the EBV of sires with less than 30 daughters: a 27% increase for sires with up to 10 daughters, 8% for sires with 11 to 20 daughters, and 5% for sires with 21 to 30 daughters. This fact contributed to the large re-ranking of the sires and the low Spearman rank correlation coefficient found.

Considering that the choice of sires to be used for breeding is the EBVs, the use of some sires may be misleading, and others should not be used. The intense use of some sires is evidenced by the low number of sires with more daughters. Therefore, without the inclusion of bioclimatic variables in the model, some sires may be penalized because their daughters are conditioned to environmental stress factors.

## CONCLUSION

The inclusion of bioclimatic variables in the genetic evaluation of Holstein dairy cattle directly affects sires selection. The ranking of sires changes severely when THI and DTV are included as fixed effects in the model, changing the EBV for milk yield and significantly improving the reliability the of EBVs of the sires.

The best way to include THI and DTV as fixed effects in the model is to consider the mean between the two last days before each TD. Considering the great diversity of environmental conditions in Brazil and that certain sires are not penalized by the environment (THI and DTV) in which their daughters are bred, it is essential to include bioclimatic variables in the genetic evaluation models to avoid compromising the genetic progress of the herds.

## Figures and Tables

**Table 1:** Summary of the standard model data structure

Item	Statistics
Animals in the pedigree file	32,409
Animals with records	11,294
Dams in the pedigree file	8,639
Sires in the pedigree file	641
Test-day records	94,549
Mean records/animal	8.37
Milk yield mean (kg/d)	25.81±7.21
Contemporary groups	5,257

**Table 2:** Reliability of estimated breeding values of the best ten sires and all sires for milk yield in the test-day (TD) records

Items	Models1)					

	SM	M7-0DB	M7-1DB	M7-2DB	M7-3DB	M7-2DM
Sires						
a	0.57	0.58	0.56	0.53	0.56	0.56
b	0.58	0.61	0.59	0.54	0.59	0.59
c	0.58	0.60	0.58	0.55	0.58	0.58
d	0.54	0.54	0.51	0.48	0.51	0.51
e	0.79	0.81	0.78	0.73	0.78	0.78
f	0.56	0.57	0.55	0.51	0.55	0.55
g	0.37	0.39	0.38	0.36	0.38	0.38
h	0.86	0.91	0.85	0.78	0.85	0.85
i	0.67	0.69	0.67	0.62	0.67	0.67
j	0.40	0.41	0.39	0.38	0.39	0.39
Means	0.59	0.61	0.59	0.55	0.59	0.59
Groups of selected sires						
Top 1%	0.52^a^ (0.27 to 0.83)*	0.48^a^ (0.27 to 0.83)	0.47^a^ (0.25 to 0.80)	0.47^a^ (0.26 to 0.80)	0.47^a^ (0.25 to 0.80)	0.56^a^ (0.38 to 0.83)
Top 5%	0.46^b^ (0.11 to 0.86)	0.42^b^ (0.11 to 0.86)	0.41^b^ (0.08 to 0.83)	0.42^b^ (0.11 to 0.84)	0.42^b^ (0.08 to 0.83)	0.51^a^ (0.24 to 0.86)
Top 10%	0.39^b^ (0.10 to 0.88)	0.36^b^ (0.10 to 0.86)	0.35^b^ (0.10 to 0.79)	0.35^b^ (0.10 to 0.84)	0.34^b^ (0.10 to 0.86)	0.46^a^ (0.11 to 0.88)
All	0.16^b^ (0.01 to 0.92)	0.15^b^ (0.01 to 0.92)	0.14^b^ (0.01 to 0.90)	0.15^b^ (0.01 to 0.90)	0.14^b^ (0.01 to 0.90)	0.28^a^ (0.05 to 0.90)

THI, temperature-humidity index; DTV, diurnal temperature variation.

1) According to the standard model (SM) and best adjusted model (M7) for the days included with THI and DTV data—on the TD record (0DB); one day before (1DB); two days before (2DB); three days before (3DB), and mean between the two last days before (2DM).

ab Median followed by different letters in the rows are significantly different (p<0.05) by the Kruskal-Wallis test.

* Minimum and maximum.

**Table 3:** Average reliability of estimated breeding values (EBVs) of the sires, according to their number of daughters; comparison between the traditional standard model (SM) and the model that includes the mean of two days of bioclimatic variables as fixed effects (M7-2DM)

N daughters	N sires	Reliability of EBVs	

		SM	M7-2DM
<10	364	0.34 (0.01 to 0.85)*	0.43 (0.05 to 0.86)
11 to 20	127	0.52 (0.34 to 0.81)	0.56 (0.35 to 0.82)
21 to 30	59	0.63 (0.51 to 0.82)	0.66 (0.51 to 0.83)
31 to 40	23	0.68 (0.58 to 0.75)	0.69 (0.61 to 0.76)
41 to 50	12	0.73 (0.67 to 0.81)	0.74 (0.69 to 0.82)
51 to 100	43	0.78 (0.68 to 0.85)	0.79 (0.69 to 0.86)
>101	13	0.86 (0.83 to 0.92)	0.87 (0.83 to 0.92)

* Minimum and maximum.
